# Asymptomatic *Clostridium difficile* colonization: epidemiology and clinical implications

**DOI:** 10.1186/s12879-015-1258-4

**Published:** 2015-11-14

**Authors:** Luis Furuya-Kanamori, John Marquess, Laith Yakob, Thomas V. Riley, David L. Paterson, Niki F. Foster, Charlotte A. Huber, Archie C. A. Clements

**Affiliations:** Research School of Population Health, The Australian National University, Building 62 Mills Road, Canberra, ACT 2601 Australia; School of Population Health, The University of Queensland, Herston, QLD Australia; Queensland Department of Health, Communicable Diseases Unit, Herston, QLD Australia; Department of Disease Control, London School of Hygiene and Tropical Medicine, London, UK; Microbiology and Immunology, School of Pathology and Laboratory Medicine, The University of Western Australia, Nedlands, WA Australia; PathWest Laboratory Medicine, Queen Elizabeth II Medical Centre, Nedlands, WA Australia; The University of Queensland, UQ Centre for Clinical Research, Herston, QLD Australia

**Keywords:** *Clostridium difficile*, Carrier state, Asymptomatic, Infection

## Abstract

**Background:**

The epidemiology of *Clostridium difficile* infection (CDI) has changed over the past decades with the emergence of highly virulent strains. The role of asymptomatic *C. difficile* colonization as part of the clinical spectrum of CDI is complex because many risk factors are common to both disease and asymptomatic states. In this article, we review the role of asymptomatic *C. difficile* colonization in the progression to symptomatic CDI, describe the epidemiology of asymptomatic *C. difficile* colonization, assess the effectiveness of screening and intensive infection control practices for patients at risk of asymptomatic *C. difficile* colonization, and discuss the implications for clinical practice.

**Methods:**

A narrative review was performed in PubMed for articles published from January 1980 to February 2015 using search terms ‘Clostridium difficile’ and ‘colonization’ or ‘colonisation’ or ‘carriage’.

**Results:**

There is no clear definition for asymptomatic CDI and the terms carriage and colonization are often used interchangeably. The prevalence of asymptomatic *C. difficile* colonization varies depending on a number of host, pathogen, and environmental factors; current estimates of asymptomatic colonization may be underestimated as stool culture is not practical in a clinical setting.

**Conclusions:**

Asymptomatic *C. difficile* colonization presents challenging concepts in the overall picture of this disease and its management. Individuals who are colonized by the organism may acquire protection from progression to disease, however they also have the potential to contribute to transmission in healthcare settings.

**Electronic supplementary material:**

The online version of this article (doi:10.1186/s12879-015-1258-4) contains supplementary material, which is available to authorized users.

## Background

*Clostridium difficile* is a Gram-positive, spore-forming, anaerobic bacillus that was first described in 1935 as part of the intestinal flora of newborn infants [1]. *C. difficile* is recognized as one of the most important pathogens in hospital and community healthcare settings, with a steadily rising global incidence of infection and concordant increase in mortality [2, 3]. The Centers for Disease Control and Prevention in the USA have assigned *C. difficile* as an urgent threat because of its association with antibiotic use and high mortality and morbidity [4].

The clinical spectrum of symptomatic *C. difficile* infection (CDI) ranges from mild diarrhea to severe complications such as pseudomembranous colitis, toxic megacolon, bowel perforation, sepsis, and death [5]. Symptomatic CDI is mediated through the production of toxins that are cytotoxic to epithelial cells of the colon, causing extensive inflammation and epithelial tissue damage to the host [6]. These toxins (toxins A and B) are implicated as the major virulence factors of *C. difficile*. An additional putative virulence factor, the binary toxin, is produced by some strains, particularly the more virulent epidemic strains such as BI/NAP1/027, and may also be present in the absence of toxin A or toxin B [7].

Asymptomatic *C. difficile* colonization is the condition where *C. difficile* is detected in the absence of symptoms of infection. It has been proposed that asymptomatic *C. difficile* colonized patients may be protected from progression to infection because they can mount a humoral immune response to clostridial toxins [8]. However, asymptomatic *C. difficile* colonized patients potentially act as an infection reservoir and may present a risk to others [9, 10]. The number of colonized patients is higher than symptomatic CDI cases among hospital patients, particularly when disease is endemic [11–13]. The prevalence of asymptomatic *C. difficile* colonization varies depending on a number of host, pathogen, and environmental factors. These features of asymptomatic *C. difficile* colonization are important to establish the contribution that asymptomatic *C. difficile* colonized patients make as potential vehicles of transmission of *C. difficile* in healthcare environments, particularly with the global spread of emergent hypervirulent toxigenic strains [14].

Few studies have synthesized evidence on the role and importance of asymptomatic *C. difficile* colonization in the progression to symptomatic CDI, the transmission of infection, or the challenges to CDI control. Therefore, we have reviewed published literature (Additional file 1) describing asymptomatic *C. difficile* colonization to better understand the prevalence, risk factors for colonization, mechanisms that may protect colonized patients from progression to symptomatic CDI or recurrent disease and the risk asymptomatic *C. difficile* colonized patients pose to non-colonized patients.

### Definition of symptomatic *C. difficile* infection and asymptomatic *C. difficile* colonization

It is generally accepted that positive assays for *C. difficile* toxins are indicative of active disease and that the toxins are responsible for clinical symptoms [15, 16]. A validation study comparing reference tests for *C. difficile* (toxin assay positive versus cytotoxigenic *C. difficile* culture positive/toxin assay negative) showed that detection of toxins was associated with more severe CDI outcomes [17]. However, it has also been reported that patients with positive toxin assays can remain symptomless [8, 10, 18]. Therefore, the sole presence of *C. difficile* toxins is insufficient for a diagnosis of the disease. Consequently, symptomatic CDI has been defined as:

- The presence of diarrheal symptoms (three or more unformed stools in 24 or fewer consecutive hours) and either
  - ○ a stool test result positive for *C. difficile* toxins or
  - ○ detection of toxigenic *C. difficile*, or
  - ○ colonoscopic findings demonstrating pseudomembranous colitis [19].

To our knowledge there is no clear definition for asymptomatic CDI and the terms carriage and colonization are often used interchangeably. Table 1 provides case definitions for asymptomatic carriage and colonization identified in this review to illustrate the heterogeneity of the definitions used by different the authors and that both terms have been used without distinction. For the sake of clarity, while maintaining conventions of previous studies, we recommend the following definition for asymptomatic *C. difficile* colonization:

**Table 1:** A description of different case definitions for asymptomatic colonization and carriage with *C. difficile*

Term used	Case definition	Study reference
Colonization	Patients with symptomless colonization were defined as symptom-free, excluding patients recovering from *C. difficile* associated diarrhea.	Shim, 1998 [8]
	Asymptomatic *C. difficile* colonization was defined as a positive stool culture for *C. difficile* in the absence of diarrhea.	Loo, 2011 [13]
	A case of toxigenic *C. difficile* colonization was defined as an asymptomatic individual with *tcdB* gene detected in a fecal sample by real-time PCR	Hung, 2012 [109]
	Was not specifically defined and did not distinguish between colonization and infection. One colonized case was symptomatic at sampling time (personal communication).	Arvand, 2012 [30]
Carriage	Asymptomatic carriage was defined as a positive stool culture or cytotoxin test and the absence of diarrhea during hospitalization and during a 30-day period after discharge.	Kyne, 2000 [18]
	Asymptomatic carriage was considered when *C. difficile* or its cytotoxin was detected in stool from persons without gastrointestinal symptoms.	Simor, 1993 [67]
	Carriers were defined as positive for a toxigenic *C. difficile* screening test during the study period in the absence of a clinician ordered toxin screen determined by electronic medical record review. Carriers were categorized as persistent, transient, or indeterminate.	Curry, 2013 [75]

- The absence of diarrhea (or if present, attributable to a cause other than CDI) without colonoscopic or histopathologic findings consistent with pseudomembranous colitis, and either
  - ○ the detection of *C. difficile* or
  - ○ the presence of *C. difficile* toxins.

Novel to this definition of asymptomatic *C. difficile* colonization is the acknowledgment that symptoms associated with CDI can arise from alternative underlying conditions. Diarrhea commonly affects hospitalized patients and in the majority of the cases is attributable to non-infectious (e.g. medication side-effects, inflammatory bowel disease) and infectious causes other than CDI [20]. The proportion of cases of nosocomial diarrhea attributable to CDI may be within the range of 20 to 25 % [21, 22]. Identification of the etiology of diarrhea (or even to rule out *C. difficile*) could be challenging, particularly in critically ill patients. In cases where the underlying cause(s) of diarrhea cannot be identified (or CDI remains as a differential diagnosis), we suggest the use of algorithms such as the one proposed by Polage and colleagues [20]. They suggested that regardless of their antibiotic exposure status, CDI should be considered in all patients with clinically significant diarrhea. The evaluation of a patient should start by verifying the presence of diarrhea; the frequency, consistency, volume of stool, and duration of diarrhea should be taken into account along with associated symptoms/signs such as cramping, dehydration, fever, hypotension, or sepsis. If no clear infectious cause is identified, the medical history must be reviewed for non-infectious or iatrogenic (e.g. laxative overdose) causes.

There is no evidence that non-toxigenic *C. difficile* strains can cause disease [23]. In studies reporting CDI from patients harboring non-toxigenic strains, the cultured organisms were mixed with toxigenic stains and could not definitively be associated with disease [24, 25]. Hence, individuals with diarrhea who test positive only for non-toxigenic strains of *C. difficile* should be considered asymptomatically colonized unless there is supporting evidence of disease, such as endoscopic findings consistent with pseudomembranous colitis. In addition, colonization can be transient or long term often depending on the extent and frequency of exposure to *C. difficile.*

### Epidemiology

Prevalence estimates of asymptomatic *C. difficile* colonization vary considerably between different patient groups (Table 2). Among healthy adults with no prior risk factors for CDI, asymptomatic *C. difficile* colonization prevalence varied between 0 and 15 % [15, 26–33]. The study reporting 15 % was a prospective cohort study carried out on seven groups of healthy individuals representing various occupations in Japan [32]. The range of asymptomatic *C. difficile* colonization prevalence among groups of study subjects was 4 to 15 %; the groups comprised university students, hospital workers, company employees, and defense force personnel. Among healthy newborns and infants, the prevalence of asymptomatic *C. difficile* colonization varied between 18 and 90 % [15, 34].

**Table 2:** Prevalence of asymptomatic *C. difficile* colonization in different populations

Population type		Range of carriage rates	References
Healthy neonates and infants		18–90 %	[34, 110–113]
Healthy adults – general population		0–15 %	[15, 26–33]
Elderly in long-term care facilities, chronic care, or nursing homes		0–51 %	[9, 30, 37, 66, 67, 70, 114–116]
Hospital	*Elderly*	0.6–15 %	[26, 68, 69, 114, 117, 118]
	*Inpatients (not specifically elderly)*	4–29 %	[10, 13, 18, 22, 73, 79, 91, 105, 106, 109, 119–121]
	*Rehabilitation (spinal)*	11–50 %	[43, 45]
	*HIV*	4 %	[122]
	*Healthcare workers*	0–13 %	[26, 32, 123]
	*Cystic fibrosis*	18–47 %	[38–41]
	*Hospital surgical patients on antibiotic prophylaxis*	17 %	[124]
	*Intensive care*	7 %	[125]
	*IBD (ulcerative colitis or Crohn’s disease)*	11 %	[95]
	*Hematological malignancies*	8 %	[94]

Few studies have examined asymptomatic *C. difficile* colonization in acute hospital care settings. In 1982, Gerding and colleagues detected 43/146 (29 %) patients colonized with non-toxigenic *C. difficile* strains [22]. Over the course of 10 years (1982–1991), Belmares and colleagues reported overall colonization with non-toxigenic strains in 10 % of the patients (ranged from 5 % in 1982 to 18 % in 1984) [35]. Most studies reporting asymptomatic *C. difficile* colonization have targeted elderly patients in dedicated long-term care facilities (LTCFs). Prevalence of asymptomatic *C. difficile* colonization among elderly residents ranged from 0 to 51 %, possibly because CDI is often endemic in units or institutions with elderly patients [9, 30, 36, 37].

Among adults, the highest prevalence of asymptomatic *C. difficile* colonization has been reported in patients with cystic fibrosis (CF) and in spinal/brain injury rehabilitation. Asymptomatic *C. difficile* colonization prevalence ranged from 18 to 47 % in studies among CF patients, substantially higher than other clinical subgroups (e.g. surgical patients) or general hospital inpatients [38–42]. In a case–control study, Bauer and colleagues found 26/55 (47 %) CF patients were asymptomatically colonized [38]. Yahav and colleagues reported 14 toxin-positive asymptomatic *C. difficile* colonized patients without evidence of diarrhea in a study of 30 CF patients compared to no toxin-positive individuals among non-CF patients [41]. Welkon and colleagues reported asymptomatic *C. difficile* colonization in 19/99 CF patients (19 %), with 12 strains being toxigenic [40]. Another study of CF patients reported asymptomatic *C. difficile* colonization in 12/37 (32 %) patients, rising to 43 % if patients were treated with antibiotics [39]. The heightened vulnerability of CF patients to asymptomatic *C. difficile* colonization rather than to disease has been attributed to an electrolyte transport defect in epithelial cells that may offer protection from the effects of clostridial toxins [41].

Rehabilitation patients also had higher asymptomatic *C. difficile* colonization prevalence than other groups. In one study, 11/22 (50 %) spinal cord rehabilitation patients were colonized and remained asymptomatic [43]. The asymptomatic *C. difficile* colonized patients in this study also had a significantly greater length of stay (median 57 days) compared to non-colonized patients (median 6 days). Stevens and colleagues found that for 7-day increments in length of stay, the risk of healthcare-associated CDI increased by 10 % [44]; this implies that on average, spinal cord rehabilitation asymptomatic *C. difficile* colonized patients will be at 52 % increased risk of developing CDI compared to non-colonized *C. difficile* patients. Another study of asymptomatic *C. difficile* colonization prevalence on admission to a rehabilitation ward reported that 9/54 (17 %) patients without prior colonization became colonized after admission [45]. Of these nine patients, six showed no symptoms of diarrhea. The increased colonization rate among this group of patients is thought to result from the rehabilitation therapy where group activities and socialization are encouraged, facilitating transmission.

#### Mechanism of colonization with C. difficile

The first stage in asymptomatic *C. difficile* colonization is the ingestion of *C. difficile* spores [46–48]. The spores survive the gastric acid and germinate into vegetative cells in the anaerobic environment of the colon. *C. difficile* has been isolated from samples of human jejunum, however the primary reservoir is the large intestine [49]. Vegetative *C. difficile* cells penetrate the mucus layer in the large intestine using flagella and enzymatic degradation of the colonic extracellular matrix [48]. Once the mucosal layer has been breached, in vitro assays have demonstrated that adhesion of *C. difficile* cells to intestinal epithelial cells is facilitated by bacterial surface layer proteins [50].

For colonization with vegetative *C. difficile* cells to occur, there must be a disruption of the normal intestinal microbiota which usually provides colonization resistance against *C. difficile* [51, 52]. The inhibitive effect of the natural gut microbiota may occur through competition for space and nutrients or the production of compounds that inhibit *C. difficile* proliferation [53]. The concept of colonization resistance is important to understand the mechanisms that result in the development of disease. Therefore, there is potential to introduce non-pathogenic organisms as probiotic agents or non-toxigenic *C. difficile* strains to compete with toxigenic *C. difficile* strains as novel prevention and treatment strategies [54, 55]. However, Brouwer and colleagues have challenged this concept as they found that transconjugation of the pathogenicity locus can occur from toxigenic to non-toxigenic *C. difficile* strains [56].

#### Toxin production and asymptomatic colonization

Secretion of toxins A and B usually occurs once *C. difficile* reaches the stationary phase. The first essential step for these toxins to exert their effects is binding to receptors on gut epithelial cells [6]. Disease symptoms commence with toxin catalysis in the cytosol. The catalyzed toxin products inactivate guanosine triphosphate binding Rho proteins [6]. The subsequent depolymerization of the actin cytoskeleton elicits a cellular response that includes neutrophil infiltration, resulting in inflammation, and the subsequent release of cytokines and interferon gamma [57, 58]. Cell death occurs by apoptosis following disaggregation of the actin cytoskeleton [59]. Consequently, extensive colonic inflammation and epithelial tissue damage occur, leading to rapid fluid loss into the large intestine, manifesting as acute diarrhea [6].

The role and importance of toxins A and B in progression to the disease state has been subject to debate. In early studies using hamster models, purified toxin A was shown to elicit symptoms consistent with disease, whereas toxin B would only elicit a response if co-administered with toxin A [60]. Consequently, it was suggested that toxin B exerted an effect following initial tissue damage by toxin A. The recovery of toxin A-negative, toxin B-positive strains from symptomatic patients has challenged the view that toxin A is the dominant virulence factor in CDI [61, 62]. Recent work with animal models using antibodies against toxins A and B showed that administration of anti-toxin B antibodies either alone or in combination with anti-toxin A was more effective at preventing the development of gastrointestinal symptoms consistent with CDI [63]. Lyras and colleagues constructed mutant isogenic strains of *C. difficile* capable of producing either toxin A or toxin B. The toxin A producing strains lost their pathogenicity whereas the toxin B producing stains were as pathogenic in animal models as wild type strains [64]. However, another group using similar gene knockout methods to generate mutant strains produced conflicting findings with a role for both toxins A and B [65].

Toxigenic strains of *C. difficile* are the most prevalent among colonized patients; early studies cultured stool specimens and using enzyme immunoassay (EIA) or cell culture cytotoxicity neutralization assay reported the proportion of toxigenic strains among asymptomatic colonized patients was in excess of 50 % [31, 39, 40, 66–69]. These findings have been corroborated in later studies using real-time polymerase chain reaction (PCR) [27, 29, 30, 32, 70]. It is important to note that both EIA and PCR methods specifically target toxigenic *C. difficile* strains and could therefore bias results reporting a higher prevalence of these strains [71].

#### Duration of the colonized state

There is limited information about the duration over which individuals remain asymptomatic after coming in contact with *C. difficile* spores or the time taken to revert to a non-colonized state. In a randomized placebo-controlled trial, Johnson and colleagues compared the efficacy of vancomycin and metronidazole for eradication of *C. difficile* in asymptomatic colonized patients. Sixty, 80 and 100 % of the patients in the placebo group were negative for *C. difficile* after 40, 70 and >90 days follow-up, respectively [72]. In a prospective study, Samore and colleagues [73] compared the incidence of colonization in surgical, medical and intensive care wards. Thirty two colonized patients were followed on a weekly basis until they were discharged; 84 % of the colonized patients remained culture positive with median duration of colonization of 8.5 days (range 7–29 days). The study also showed that 3/20 (15 %) of the patients colonized with non-toxigenic strains, none of whom developed diarrhea, were positive for toxigenic strains at follow-up. Longer-term colonization and transmission was investigated among 1234 healthy Japanese volunteers, who included university students, hospital staff, and company employees [32]. Follow-up was performed on 38 asymptomatic patients between 5 and 7 months later. Of these 38 cases, *C. difficile* was re-isolated from 12 (32 %) individuals, half of whom yielded the same PCR ribotypes and pulsed-field gel electrophoresis types as previously. In a subsequent study by the same authors, a 6-month follow-up of 18 colonized healthy students found 10 (56 %) were no longer colonized and 8 (44 %) were colonized more than once, of whom 3 (38 %) harbored the same strain [27].

These findings suggest that there is marked variation in duration of the colonized state, however the role of repeated exposure from the environment or other colonized individuals was not investigated. Limited longitudinal data available about asymptomatic *C. difficile* colonization warrants further epidemiological studies to investigate the persistence of colonization and to understand the role of re-exposure to the organism over time.

#### Transmission from colonized patients

Person-to-person transmission in hospital wards, environmental contamination, and carriage of *C. difficile* on the hands of healthcare workers have been described extensively [74–77]. The main modes of transmission are by the fecal-oral route and direct contact with contaminated surfaces and fomites [78], although transmission between healthy individuals who are asymptomatically colonized has also been reported [32].

Spores from asymptomatically colonized patients are a potential source of CDI and may contribute to the transmission reservoir [9] and studies have clearly demonstrated that transmission from asymptomatically colonized patients can occur [75, 79]. Curry and colleagues investigated transmission potential of asymptomatic *C. difficile* colonized patients using multiple-locus variable number tandem repeat analysis. They found that 29 % of isolates from hospital-associated CDI cases were highly related to isolates from asymptomatic *C. difficile* colonized patients [75]. Clabots and colleagues reported that patients admitted from home without prior hospitalization in the previous month had the lowest prevalence of asymptomatic *C. difficile* colonization (6 %) but, because they represent the majority of admissions, they contributed the second-highest total number of *C. difficile* introductions to hospital, after patients readmitted to hospital within 30 days [79]. Similarly, the length of stay in hospital can also influence transmission. Fecal excretion of *C. difficile* spores occurs for up to 6 weeks following resolution of CDI symptoms [80, 81]. Furthermore, Riggs and colleagues demonstrated that even colonized patients who did not develop disease during a 6 months follow-up period were shedding spores into the environment [9]. The current CDI clinical practice guidelines from the Society of Healthcare Epidemiologists of America (SHEA) recommend maintaining contact precautions only until resolution of diarrhea. It has been suggested that contact precautions should be extended until time of discharge for patients recovering from CDI. However, there is no conclusive evidence to support extending contact precautions following CDI while patients remain asymptomatic during their hospital stay [81].

Asymptomatic *C. difficile* colonized patients in hospital have the potential to contaminate the environment and subsequently infect others [75]; however the transmission potential is lower in asymptomatic *C. difficile* colonized patients than in those patients with active disease [10]. In one prospective study of acquisition rates in an endemic CDI setting, 38/128 (29 %) environmental samples from hospital rooms occupied by asymptomatic *C. difficile* colonized patients were contaminated compared to 90/128 (49 %) samples from rooms occupied by patients with disease. This corresponds with findings from another study of LTCF residents in which proportions of positive cultures from skin sites and environmental samples were highest among residents with disease, second highest among asymptomatic *C. difficile* colonized patients and lowest among non-colonized residents [9]. Moreover, Sethi and colleagues found that even 4 weeks after receiving therapy for CDI, the frequency of skin contamination (30/52; 58 %) and environmental shedding (26/52; 50 %) remained high in asymptomatic *C. difficile* colonized patients [81]. Samore and colleagues demonstrated that in an endemic situation carriage of *C. difficile* on the hands of healthcare workers was positively correlated with the extent of environmental contamination with *C. difficile* [82].

The spore-forming ability of *C. difficile* makes it distinct from other infectious organisms common to healthcare settings and introduces further challenges to reduce transmission. Spores can persist in the environment for long periods and require chlorine- [83] or peroxide-based [84] sporicidal agents or ultraviolet radiation devices [85] for environmental decontamination. Typically, hospital patients colonized with other multidrug-resistant organisms are isolated to prevent transmission, but this appears to be of limited value for asymptomatic *C. difficile* colonization. In an epidemiological model, Lanzas and colleagues demonstrated that transmission of *C. difficile* within a ward cannot be sustained unless new *C. difficile* colonized patients are introduced [86]. Therefore, the admission of asymptomatic *C. difficile* colonized patients plays an important role in sustaining *C. difficile* transmission within a ward [87]. A recent study, has demonstrated that nearly half of the *C. difficile* cases were genetically distinct from all previous cases, which suggests genetically diverse sources of infection [88]. Furthermore, Yakob and colleagues demonstrated, using a stochastic mathematical model, that screening for asymptomatic *C. difficile* colonization to segregate colonized patients from non-colonized patients had little impact on infection transmission because patients still in a latent period (exposed but not yet colonized) would not be detected [89].

### Risk factors for asymptomatic *C. difficile* colonization and progression to active disease

Among inpatients with positive stool samples for *C. difficile*, McFarland and colleagues found that 52/83 (63 %) of the patients were asymptomatic and 31/83 (37 %) developed symptoms of CDI [10]. Currently, the time required to progress from asymptomatic *C. difficile* colonization to active CDI is unknown; however, epidemiological studies have identified risk factors associated with progression to disease. It is not surprising to find common risk factors for asymptomatic *C. difficile* colonization and disease because colonization with *C. difficile* is a necessary prerequisite of disease. The most significant epidemiological study to date to investigate risk factors for healthcare-associated asymptomatic *C. difficile* colonization identified that hospitalization within the last 12 months, exposure to corticosteroids, history of CDI and presence of antibody against toxin B were significantly associated with healthcare-associated asymptomatic *C. difficile* colonization [90]. Similar findings were described by Loo and colleagues in 2011, they identified chemotherapy, recent hospitalization, use of proton-pump inhibitors or histamine H2 antagonists, and presence of antibodies against toxin B were associated with healthcare-associated asymptomatic *C. difficile* colonization [13]. The study also found that antibiotic exposure (within 8 weeks of hospitalization) was as a risk factor for healthcare-associated CDI (OR 5.25, 95 % CI 2.15–12.82) but not for healthcare-associated asymptomatic *C. difficile* colonization (OR 1.04, 95 % CI 0.61–1.78). The apparent discrepancy between the results may indicate that disruption of the intrinsic intestinal microbiota due to antibiotic exposure is not a key feature for *C. difficile* colonization as it is for progression to disease. More recently, an investigation conducted in a tertiary care facility identified recent hospitalization, chronic dialysis, and corticosteroid use as independent risk factors for toxigenic asymptomatic *C. difficile* colonization on admission [91]. The eligible patients’ first stool samples after admission were tested for toxigenic *C. difficile* by real-time PCR assay. While the study had limited generalizability, because the subjects who participated in the study were predominantly older (mean age 64 years), and due to the low proportion of enrolled subjects who provided samples (22 %), results were consistent with a previous study that reported renal disease, prior hospital admission, and prior CDI as risk factors for culture positivity on admission [73].

**Fig. 1:**
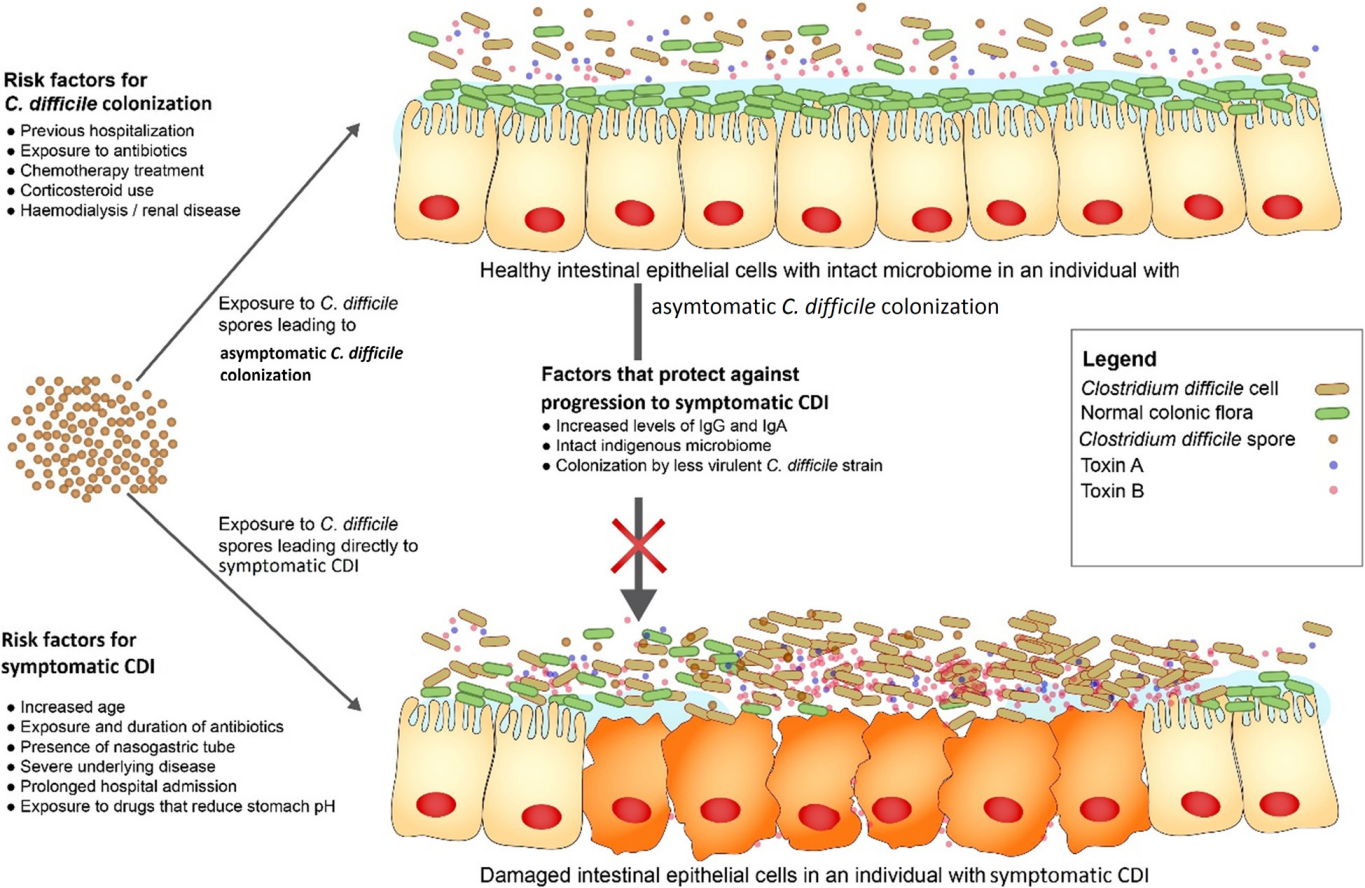
Diagrammatic representation of asymptomatic *C. difficile* colonization and progression to symptomatic *C. difficile* infection (CDI). Following exposure to *C. difficile* spores, an individual may transiently or persistently harbor the organism without expressing typical CDI symptoms. In other situations ingestion of *C. difficile* spores or vegetative cells may lead directly to symptomatic CDI. Some individuals with asymptomatic *C. difficile* colonization may progress to symptomatic CDI, however a number of host and pathogen related factors can limit the progression of the disease

There are limited data about risk factors for asymptomatic *C. difficile* colonization among healthy populations. McNamara and colleagues investigated environmental factors associated with an increased risk of asymptomatic *C. difficile* colonization in a cohort of healthy farm workers. They found that reported weekly exposure to lake or pond swimming was associated with asymptomatic *C. difficile* colonization [29]; although, no biological plausible explanations were given for this finding by the authors. A number of factors act in concert before asymptomatic *C. difficile* colonization progresses to active disease. These factors can be categorized as host mediated or pathogen related. A diagrammatic representation of the mechanism of asymptomatic *C. difficile* colonization and progression to disease with risk factors is shown in Fig. 1.

#### Host-mediated factors

The most significant factor that leads to CDI is the disruption of intrinsic colonization resistance. This is a feature of the human intestine whereby indigenous microbiota, and the presence of compounds that inhibit bacterial germination and proliferation protect individuals against diseases caused by pathogenic organisms [54]. Factors that disrupt the intestinal microbiota thereby allowing *C. difficile* to flourish include treatment with antibiotics, proton-pump inhibitors, and chemotherapy agents in addition to physical effects of abdominal surgery and nasogastric tubes [13, 92].

Other host factors associated with an increased risk of CDI include advanced age, multiple comorbidities, suppressed immune system, inflammatory bowel disease and dense intestinal co-colonization with enterococci [27, 31, 69, 93–95]. It is worth pointing out that the observed association between advanced age and multiple comorbidities infection, and the increased risk of CDI, may be confounded by medication exposure given that polypharmacy is common among these groups of patients.

There is substantial evidence that asymptomatic *C. difficile* colonization has a protective effect against progression to disease through an immune-mediated response. In a prospective study of hospital patients showed that at the time of colonization, IgG levels were higher in asymptomatic *C. difficile* colonized patients compared to patients who subsequently developed diarrhea [18]. The same authors demonstrated that patients with a single episode of diarrhea had increased IgM levels against toxins A, B and non-toxin antigens compared to patients with recurrent disease, indicating that the presence of these antibodies conferred a protective effect [96]. Many healthy children and approximately 60 % of adults have detectable serum IgG and IgA antibodies to *C. difficile* toxins A and B, even when the organism is not detected [97, 98]. If antibodies are stimulated during infancy and through further exposure to *C. difficile* from the environment [99], it would suggest that protection against CDI is a dynamic host-mediated characteristic [18, 100]. The control of toxin-induced intestinal inflammation by up-regulation of A_2B_ adenosine receptors in the intestinal epithelium can also reduce the progression of aggressive symptoms of disease [101]. In this study, an A_2B_ adenosine receptor antagonist did not reduce fecal toxin levels in animal models but conferred protection against progression of disease.

#### Pathogen factors

Colonization with non-toxigenic strains of *C. difficile* can offer protection against infection, suggesting a possible colonization resistance role through competition for nutrients or access to mucosal epithelial cells [55, 102]. Competition between clostridial strains may reduce the proliferation of pathogenic strains and the onset of disease symptoms [103]. Initial speculation was that toxigenic *C. difficile* strains may be in the minority among asymptomatic *C. difficile* colonized patients [104]; however, it has since been shown that the majority of strains are toxigenic.

## Discussion and conclusion

Despite technological advances in *C. difficile* microbiology and epidemiology (e.g. genotyping), asymptomatic *C. difficile* colonization remains as a complex and challenging health problem as its epidemiological features vary considerably between study groups and settings. Several gaps in the current knowledge were identified in this review that should guide future research studies:

1. There is no consistent definition for asymptomatic *C. difficile* colonization; a standard definition across studies is urgently needed.
2. The time between acquisition of *C. difficile* and symptomatic disease is unknown but has been estimated to be between 1 and 2 weeks [8, 13, 105]. It has also been suggested that progression to disease happens within this short time after acquisition or does not occur at all [73].
3. Asymptomatic *C. difficile* colonized patients serve as a potential infection reservoir of horizontal transmission of *C. difficile* in a range of healthcare settings and the strain types isolated from patients with asymptomatic *C. difficile* colonization are predominantly toxigenic [9, 27, 30, 32, 40, 66, 70, 73, 91, 106]. However, whether the clinical outcomes differ in asymptomatic patients colonized with toxigenic *C. difficile* compared to non-toxigenic strains it is currently unknown; thus, we suggest that patients with diarrheal symptoms with non-toxigenic strains of *C. difficile* should be considered colonized unless there is definitive evidence of disease.
4. Estimates of asymptomatic colonization may be underestimated as stool culture is not practical in a clinical setting; however, this constitutes important future epidemiological study.

The current SHEA guidelines for CDI recommend that active screening for asymptomatic *C. difficile* colonization is not performed for infection control purposes [19]. Polage and colleagues retrospectively reviewed 6121 records of toxin negative patients and revealed that only one (0.02 %) had a laboratory confirmed complication of CDI. We emphasize that this recommendation for asymptomatic *C. difficile* colonization is still valid for the following important reasons: first, there are limited options to manage asymptomatic *C. difficile* colonized patients - they should not be treated because antimicrobial therapy does not eradicate spores [19, 72]; moreover treatment may render patients more susceptible to symptomatic CDI [107]; and second, asymptomatic *C. difficile* colonization might protect individuals from progressing to active diseases [8].

Given the transmission potential of asymptomatic *C. difficile* colonized patients, the increased prevalence among certain clinical groups, limited management options, and the limited utility of screening, we suggest a more pragmatic approach. Intensive infection control practices, normally reserved for diseased patients, should be targeted at individuals or clinical areas with higher risk of asymptomatic *C. difficile* colonization. For example, patient or unit-level risk assessments could target enhanced environmental cleaning and use of gloves for patient contact to limit the transmission of *C. difficile* from asymptomatic *C. difficile* colonized patients [108]. Empirical research should be conducted into the impact of targeted, risk-based, intensive infection control programs before changes to the current SHEA guidelines for asymptomatic *C. difficile* colonized patients are considered.

## Additional file

Additional file 1:
**Search strategy and selection criteria.** (DOCX 100 kb)
