# Supplementary material for: Asymptomatic Clostridium difficile colonization: epidemiology and clinical implications
Source: BMC Infect Dis. 2015 Nov 14;15:516. doi: 10.1186/s12879-015-1258-4 (PMC4647607; doi:10.1186/s12879-015-1258-4)
Supplement: Additional file 1: — Search strategy and selection criteria. (DOCX 100 kb) [file 12879_2015_1258_MOESM1_ESM.docx]

**Search strategy and selection criteria**

References for this review were identified through searches of PubMed for articles published from January 1980 to February 2015 using search terms ‘*Clostridium difficile*’ and ‘colonization’ or ‘colonisation’ or ‘carriage’.

Search details: ((((("clostridium difficile"[MeSH Terms] AND "asymptomatic diseases"[MeSH Terms]) AND colonization[All Fields]) OR colonisation[All Fields]) OR carriage[All Fields]) AND "loattrfull text"[sb] AND English[lang])

The inclusion of studies was restricted to full-text articles written in English. Conference presentations and abstracts were excluded. Articles resulting from the search and relevant references cited in those articles were reviewed by LFK and JM. All the citation were initially screened by title and abstract, the full text version of the articles that met the inclusion criteria were then reviewed.

The search identified 11 489 publications. After screening the publications by title and abstract, 11 263 were excluded. Subsequently, full-text review of 226 publications was conducted, 125 met the eligibility criteria and were included in the review (Figure S1).

| 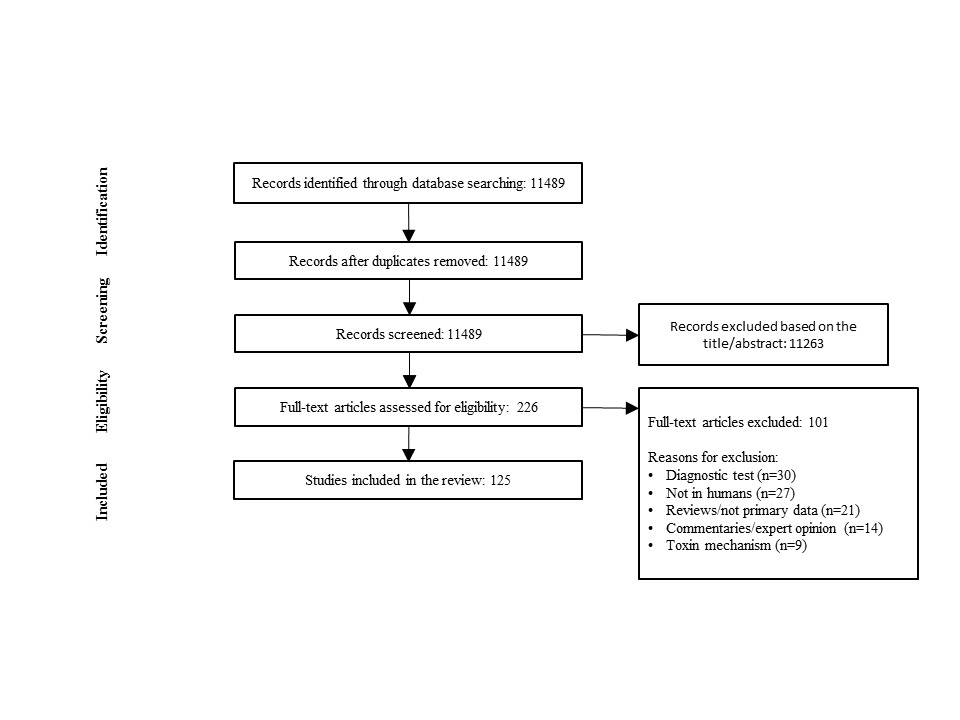 |
| --- |
| Figure S1.- PRISMA flow diagram. |
